# Piezoelectric Scaffolds as Smart Materials for Neural Tissue Engineering

**DOI:** 10.3390/polym12010161

**Published:** 2020-01-08

**Authors:** Angelika Zaszczynska, Paweł Sajkiewicz, Arkadiusz Gradys

**Affiliations:** Institute of Fundamental Technological Research, Polish Academy of Sciences, Pawinskiego 5b St., 02-106 Warsaw, Poland; psajk@ippt.pan.pl (P.S.); argrad@ippt.pan.pl (A.G.)

**Keywords:** neural tissue engineering, piezoelectric scaffolds, smart materials, polymers

## Abstract

Injury to the central or peripheral nervous systems leads to the loss of cognitive and/or sensorimotor capabilities, which still lacks an effective treatment. Tissue engineering in the post-injury brain represents a promising option for cellular replacement and rescue, providing a cell scaffold for either transplanted or resident cells. Tissue engineering relies on scaffolds for supporting cell differentiation and growth with recent emphasis on stimuli responsive scaffolds, sometimes called smart scaffolds. One of the representatives of this material group is piezoelectric scaffolds, being able to generate electrical charges under mechanical stimulation, which creates a real prospect for using such scaffolds in non-invasive therapy of neural tissue. This paper summarizes the recent knowledge on piezoelectric materials used for tissue engineering, especially neural tissue engineering. The most used materials for tissue engineering strategies are reported together with the main achievements, challenges, and future needs for research and actual therapies. This review provides thus a compilation of the most relevant results and strategies and serves as a starting point for novel research pathways in the most relevant and challenging open questions.

## 1. Introduction

The nervous system is the most complicated system in the body affecting the sensory and motor functions, when the system is damaged. Injuries of the central nervous system (CNS), i.e., brain and spinal cord, lead usually to permanent disability due to severe limitations for spontaneous regeneration of the CNS [1,2,3,4,5,6,7,8,9], leading to considerable socio-economic problems. For instance, 577 cases of traumatic brain injuries (TBI) per 100,000 people per year occurred in the U.S. alone, while, in Europe, the number of patients with diagnosed TBI was estimated at 262 per 100,000 [10]. Spinal cord injuries (SCI) are predominantly associated with irreversible loss of motor functions. In the U.S., 39 per 100,000 people were estimated to be SCI victims every year, mostly due to traffic accidents, jumping in pools, and falling from heights, while in Europe, the amount of SCI cases was 15 per 100,000 per year [11]. Regeneration of damaged neural tissue is often hindered by the presence of internal factors, such as tumor and scar tissue formation, which blocks its reconstruction. Up to date, there is no effective therapy for TBI and SCI, and surgery is able to inhibit lesion spreading only, while drug therapies have been so far focused mostly on pain relief. Hence, the current goal of CNS tissue engineering is to design a biomaterial enabling the effective outgrowth and differentiation of neural stem cells [12,13,14].

In recent decades, there has been increasing interest in research related to the development of smart materials [15]. Such materials are generally designed to respond to external stimuli (physical, chemical, mechanical) and behave similar to natural body tissues. One type of such smart materials is piezoelectric scaffolds, which can generate electrical signals in response to the applied stress [16]. Furthermore, they can stimulate the signaling pathways and thereby enhance the tissue regeneration at the impaired site. This applies especially to neural tissue, where the electrical charges are crucial for cellular activity. The major advantage of such piezoelectric scaffolds is that electrical potential can be generated non-invasively under the influence of mechanical forces, without the need to use invasive electrodes [17,18]. 

It is known that obtaining piezoelectric scaffolds is possible using various paths, involving solvent casting, TIPS (thermally induced phase separation), freeze drying, or solution blowing [19]. One of most simple and effective methods of fabricating scaffolds in the form of ultrafine fibers with diameters ranging from a few nanometers to several micrometers is electrospinning. This relatively new method uses electric force applied in the form of a very high electrostatic field to draw charged threads from polymer solutions or melts up to the fiber. The process of electrospinning depends on various parameters, which are usually divided into three groups related to the process, material, and ambient parameters. This quite simple and inexpensive technique enables the formation of nano and submicron fibers, the properties of which differ substantially from the ones observed in the bulk materials [20]. 

For neural tissue engineering, a broad spectrum of synthetic and natural polymers has been studied in the form of electrospun scaffolds [21,22,23,24,25,26,27,28,29,30]. Nerve regeneration is a localized and complex biological phenomenon that makes the treatment of patients suffering from nervous system injuries difficult. Therefore, the application of piezoelectric polymers as nerve guidance conduits allows direct delivery of electrical stimulation of the cell’s ingrowth with its electrical activity during mechanical deformation without the need for an external power source. Recent investigations have proven that neurons are extremely sensitive to electrical signals [30,31,32,33,34,35,36,37,38,39].

The aim of this paper is to summarize the studies on piezoelectric materials used for neural tissue engineering. The most used materials for neural tissue engineering strategies are reported together with the main achievements, challenges, and future needs for research and actual therapies. This review provides thus a compilation of the most relevant results and strategies and serves as a starting point for novel research pathways in the most relevant and challenging open questions.

## 2. Mechanotransduction and Piezoelectricity in Living Organisms

**Figure 1:**
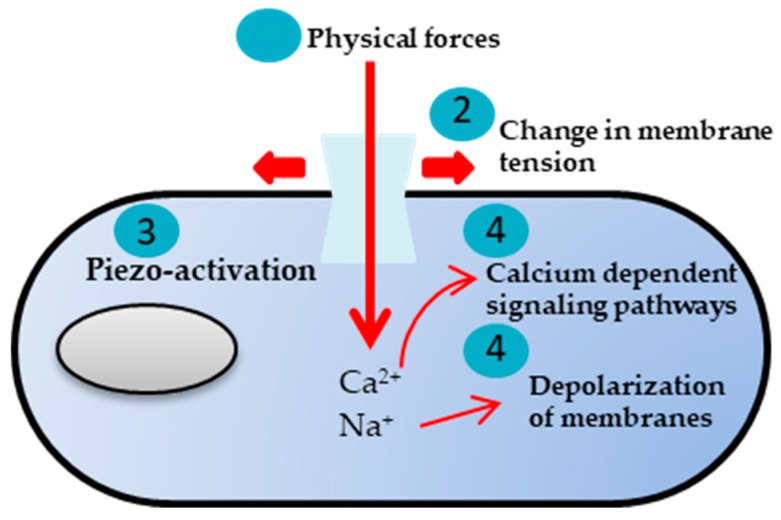
Various mechanical stimuli exerted on the cell induce changes in plasma membrane tension, eliciting piezo-channel openings (adapted from [59]).

Mechanotransduction is any of the various mechanisms through which cells sense and convert mechanical stimuli into electrochemical activity. The well known direct element on the way between external stress transmitted through the extracellular matrix and the cells are s.c. stress activated channels, being membrane proteins capable of responding to mechanical stimuli, i.e., opening or closing, generating selective ion fluxes inside the cell, resulting in a cascade of signaling processes [40,41,42,43]. Other types of transmembrane ion channels are voltage gated channels, which are activated by changes in the electrical membrane potential near the channel (Figure 1) [44,45,46]. They play a key role in excitable cells such as neuronal and muscle tissues, allowing rapid and coordinated depolarization in response to triggering voltage change. Found along the axon and at the synapse, voltage gated ion channels directionally propagate electrical signals. Voltage gated ion channels are usually ion specific, for instance to sodium (Na+), potassium (K+), calcium (Ca2+), and chloride (Cl−) ions. In the case of voltage gated channels, the natural piezoelectricity of the body elements is important for the generation of electrical charges under mechanical stress [47,48,49,50].

Mechanotransduction influences many aspects of biological functions. Nerves and neural stem cells (NSC) are sensitive to their surrounding environment, and they interact with this environment through cell surface receptors. Niche features such as substrate bound molecules, extracellular matrix (ECM) proteins, and properties such as stiffness and topography affect cell adhesion, survival, proliferation, migration, morphology, and differentiation [51,52,53,54,55,56,57,58].

**Figure 2:**
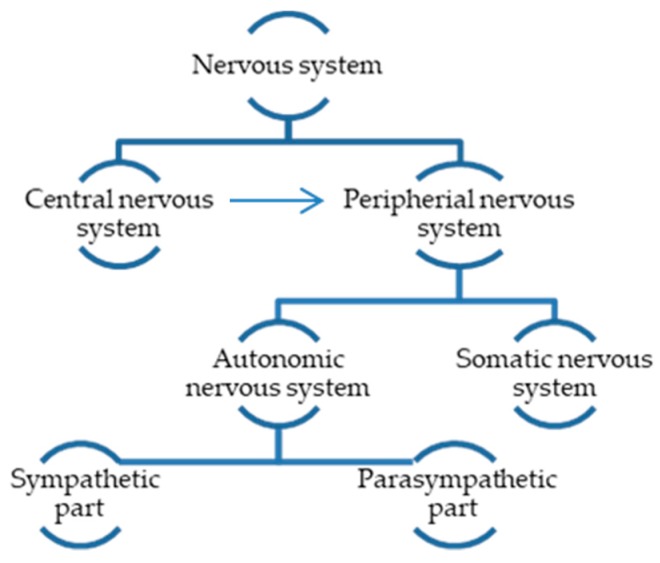
Classification of the nervous system.

The unique features of the nervous system present challenges to bioengineering research addressing nerve injuries. The nervous system is classified into the central nervous system (CNS) containing brain and spinal cord and the peripheral nervous system (PNS) created by the nerves leaving the CNS (Figure 2). The PNS somatic system transmits sensory and motor information for the CNS, while the autonomic one controls automatic functions (e.g., heart beating, blood pressure) [60,61,62].

**Figure 3:**
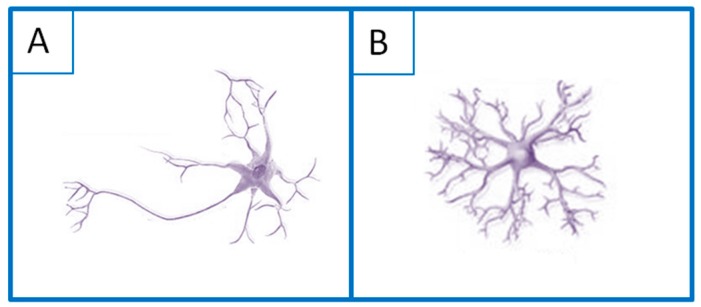
Schematic illustration of the basic units of the nervous tissue: (**A**) neuron and (**B**) neuroglia.

The basic functional units in the nervous system having specific electrical properties are neurons and neuroglia (Figure 3), which enable effective transmission of the signals [63,64]. The plasma membrane in a non-excited state is characterized by the resting potential, the value of which is usually around −70 mV [65,66,67].

**Figure 4:**
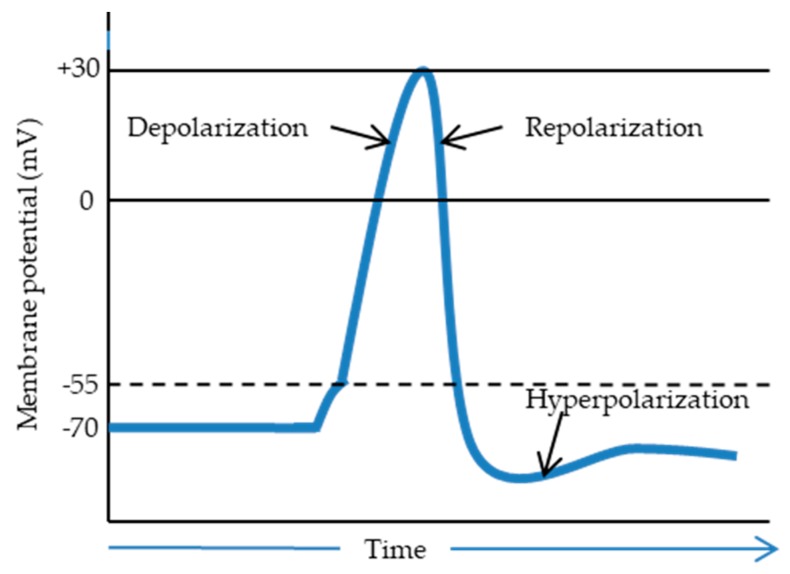
Potential difference in neural transmission as a function of time (adapted from [69]).

The electrical properties of the neural cells are described by transmission of electrical signals. This phenomenon strongly affects cell behavior via activated ion influx/efflux across the cell membrane. Another phenomenon connected with the transmission of the signals in nerve cells is the action potential. This potential can be dispersed along the axon, which next releases the neurotransmitter (presynaptic ending), and the potential is spread further. A method to activate the action potential is the application of an electrical signal across the neuron. When a dendrite receives an electrical stimulus, the Na+ channels open, and the potential changes from −70 mV to −55 mV. When the potential changes up to +30 mV, the depolarization process starts. Next, the Na+ channels close, and the K+ ones open, completing the depolarization process. When the potential reaches a value around −90 mV, hyperpolarization begins, and the next step is the repolarization of the membrane, which allows receiving another stimulus through the neuron (Figure 4). After the hyperpolarization, the Na+ and K+ channels restore the potential state at the level of −70 mV [68].

**Figure 5:**
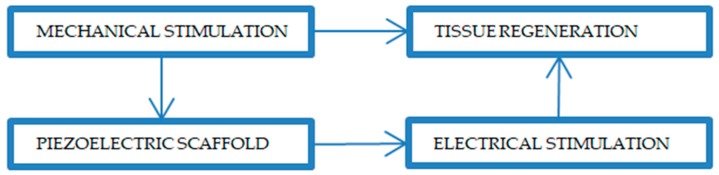
Representative scheme of tissue regeneration in response to the mechanical and electrical stimulation on the piezoelectric scaffold.

Signal transmission is the integral objective of neurons; hence, they are influenced by electrical stimuli. Many research groups have tried to explain the effect of electric stimulation on nerve regeneration. One group described the general effect of electrical stimulation on neurons [70]. Another group studied the activation of the growth controlling transport processes across the plasma membrane and the electrophoretic accumulation of the surface molecules responsible for neurite growth or cell substratum adhesion [70]. Freeman et al. [71] suggested that changes in ionic currents comprise a possible phenomenon that can affect nerve cells, while another thesis [72] described the effect of electrical stimulation on the synthesis of protein and stimulation of the neurite outgrowth in vitro. Furthermore, the authors in [73] postulated that pheochromocytoma in rat neuronal cells (PC12 cells) was electrically activated, while in [74], it was shown that the electrical stimulation increased the adsorption of fibronectin, which explains the enhanced neurite extension on electrically stimulated polypyrrole films. Additionally, nerve cells in the presence of electrical stimulation showed an extensively elongated morphology. In general, tensile/compression forces acting on the piezoelectric scaffolds generate the electrical stimulation and transfer it to the surrounding cells, promoting the cell signaling pathways, responsible for growth factor synthesis (Figure 5) [75].

**Figure 6:**
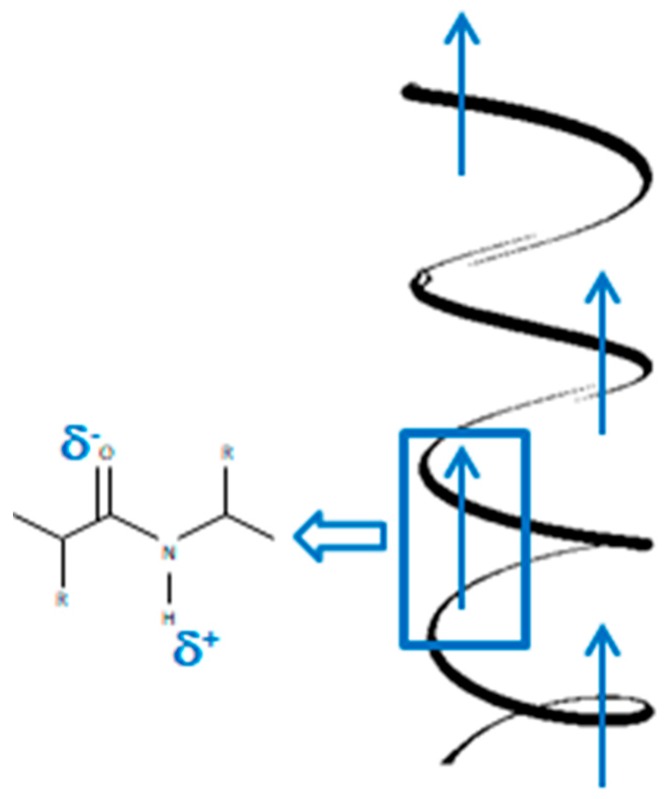
Scheme of permanent polarization in the α-helix.

In 1940, Martin [76] reported the first piezoelectric phenomena in biological tissues, when he observed electric potentials from a bundle of wool compressed by two brass plates. The main component of hair, horns, and wool is keratin in the form of an alpha-helix. It is known that the piezoelectricity of this tissue is due to the highly ordered arrangement and natural polarization of the alpha-helices, which are stabilized by hydrogen bonds between the hydrogen in the amine group and the oxygen in the carbonyl group [77,78,79,80,81,82] (Figure 6).

The results of investigations on the piezoelectric properties of biological tissues have been extensively reported [83,84,85]. The piezoelectric phenomenon has been confirmed in a variety of biological tissues, such as bone and tendons, due to the presence of highly ordered crystalline fibrillar structures such as collagen, chitin, and elastin [86,87,88,89,90]. 

**Figure 7:**
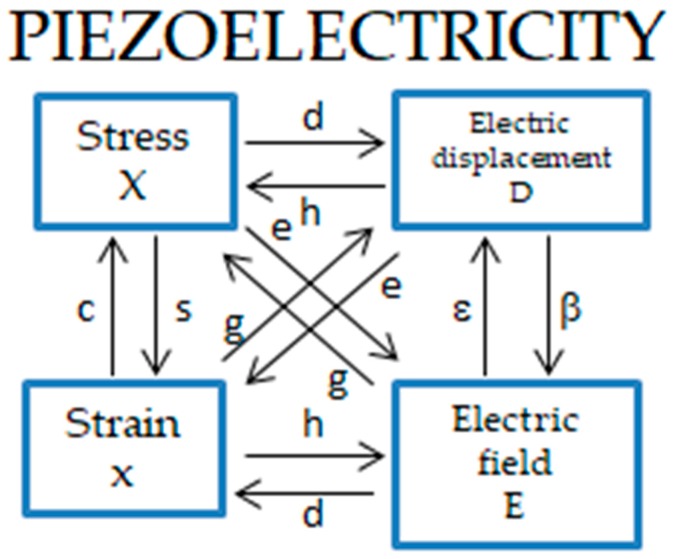
Definition of the piezoelectric coefficients (adapted from [91]).

The piezoelectric effect, by definition, is described by four piezoelectric coefficients d, e.g., and h:

(1) d = (δD/δX)^E^ = (δx/δE)^X^

(2) e = (δD/δx)^E^ = −(δX/δE)^x^

(3) g = −(δE/δX)^D^ = (δx/δD)^X^

(4) h = −(δE/δx)^D^ = −(δX/δD)^x^

which relate the electrical variables: D (electric displacement) and E (electric field) with the mechanical variables: X (stress) and x (strain). The first terms in Equations (1)–(4) describe the direct piezoelectric effect, while the second terms the converse piezoelectric effect. The subscripts relate to zero constraints of E, D, X, or x. Moreover, each of the coefficients d, e, g, and h is a third rank tensor expressed as a 3 × 6 matrix. The piezoelectric coefficients are indicated in the scheme in Figure 7, along with the coefficients *ε* (dielectric permittivity) and *c* (elastic constant) relating to each other the electrical variables D and E as well as the mechanical variables X and x, respectively.

The ratios of the coefficients *ε* and c at zero constraints of x, X, E, and D, as indicated by superscripts, define the electromechanical coupling coefficient, K, as given by:

(5) ε^x^/ε^X^ = c^E^/c^D^ = 1 − K^2^

which characterizes the efficiency of the conversion of mechanical energy to electrical energy and vice versa in the direct and converse piezoelectric effect, respectively. The piezoelectric coefficients may be determined through the direct and converse effects or the piezoelectric resonance, providing directly d, e, g, and h coefficients or the efficiency coefficient K, respectively. The results are complementary, as they cover the low (below audio) and the high frequency ranges (above 10 kHz) [91].

The piezoelectric effect is exhibited in all of the amino acid crystals, excluding alpha glycine alone. The piezoelectric coefficients found in biological materials are generally low, typically in the range of 0.1–10 pm/V (the converse effect) with the values for collagen as low as 0.2–2.0 pC/N (the direct effect). The piezoelectric properties for the most relevant natural materials are reported in Table 1.

## 3. Scaffolds: Stimuli Responsive (Piezoelectric) vs. Passive

It is generally perceived that the tissue engineering scaffolds should mimic natural existing extracellular matrix (ECM), having similarities as much as possible to the native tissues they are intended to replace in terms of the chemical composition, morphology, physical and mechanical properties, as well as biocompatibility and biodegradability. In detail, the fundamental requirements, that need to be taken into account during scaffold designing are: scaffold biocompatibility, appropriate degradation time in the case of biodegradable materials, the presence of interconnected pores in an appropriate size range, scaffold thickness, mechanical properties, and convenience of use during a surgical procedure [95,96].

It is well known that ECM is a highly dynamic structure; it is constantly being remodeled, either enzymatically or non-enzymatically, and its molecular components are subjected to various modifications. However, most of the recent artificial scaffolds remain passive, i.e., non-stimuli responsive to the external changes of the environment (static scaffolds) [97]. Moreover, conventional static scaffolds, even conductive, largely disturb the natural signaling pathways, due to their rigidity towards the signal conduction. Thus, there is a high need for smart, stimuli responsive scaffolds, which can generate and transfer the bioelectric signals analogously to the native tissues for appropriate physiological functions. Piezoelectric materials can generate electrical signals in response to the applied stress, which can be imposed even by attachment and migration of cells or body movements [98]. Using piezoelectric materials as tissue engineering scaffolds enables electrical stimulation without the need for electrodes, an external source of electricity, or implanting batteries. Such scaffolds should possess proper architecture and mechanical properties in addition in order to support cell adhesion, proliferation, and differentiation. The size of pores should be controllable and adjusted in order to enable diffusion of the metabolite, but also for appropriate cell adhesion to the biomaterial. Ninety percent porosity and a pore size in the range of 10–100 µm seem to be the most suitable for neuron growth. Scaffolds characterized by 85–90% porosity may be obtained by the electrospinning technique [99]. Such porosity is reported as supporting cellular migration and controlled diffusion of cells, metabolites, and medium, being important for cells organization, differentiation, and survival [100,101].

Obtaining a scaffold that would be biocompatible, biodegradable, conducting, and resistant to infection in order to provide neurite outgrowth is a complex task [102]. Successful nerve regeneration requires tissue engineered scaffolds not only for mechanical support of growing neurites and impediment of the ingrowth of fibrous scar tissues, but also to send biological signals to guide the axonal growth cone to the distal stump. Polymers are in general that materials that have been extensively used for creating suitable scaffolds for neural tissue [103].

Table 2 summarizes the main works on the application of piezoelectric potential scaffold materials and their applications.

## 4. Application of Piezoelectric Biomaterials in Neural Tissue Engineering

### 4.1. Piezoceramics

The earliest studied piezoelectric material group is the piezoceramics. The first applications were dated since around 1950, and since then, they have been widely used in the industry [133]. Wersing et al. [134] conducted the pioneering study on porous piezoceramics, as well as provided the basics in the theory and initial measurements [135]. Currently, there is a great need for lead-free piezoelectric materials, but the most practical ceramics are still based on lead zirconate titanate. Rat cortical neurons cultured on PZT slides coated with poly-L-lysine grew significantly longer axons, despite a decrease in cell number. Furthermore, the frequency and amplitude of the excitatory postsynaptic currents increased, suggesting that piezoelectricity could have augmented neuronal activity [136]. It is worth mentioning that piezoceramics are used for medical applications, especially medical actuators, transducers, and sensors. Due to allergic reactions, piezoceramics are not used in pure solution for medical implants. The innovative systems for medical applications are composites based on polymer matrices, with ceramic fillers, in the form of fibers [137,138,139]. To achieve bone defect repair, Lopes et al. [140] incorporated barium titanate nanoparticles into polymer matrix, which induced relatively high spontaneous polarization. Moreover, this system is characterized by reduced fragility and can be used as electroactive scaffolds [141].

#### 4.1.1. Barium Titanate 

The first piezoelectric effects in ceramics were discovered during poling of BT and led to the wide use of this material group, also as an addition in scaffolds, especially in medical applications [142,143]. Piezoceramics based on barium titanate exhibit low toxicity compared to lead based piezoelectric materials. For their high strain, they are among the most investigated groups of piezoceramics. BT nanoparticles have demonstrated cytocompatibility, even at higher concentrations of 100 μg/mL [144]. Ciofani et al. [145] demonstrated that PLGA matrix with the addition of BT nanoparticles supports the cell proliferation and attachment of osteocytes and osteoblasts. Additionally, the incorporation of barium titanate nanoparticles into the polymeric matrix improves the mechanical properties of the composite scaffold [146] and promotes cellular activity in tissue engineering applications [147].

#### 4.1.2. Boron Nitride

Boron nitride (BN) based nanomaterials play a significant role in nanotechnology owing to their conductivity, mechanical strength, and high thermal stability [148,149]. The most known boron nitride piezo-materials are in the shape of nanotubes, and with increased cytocompatibility, they can be used in tissue engineering [150] and drug delivery, due to their high piezoelectric properties [151,152,153,154,155]. It has been proven that boron nitride nanotubes have a positive influence on the adhesion of cells [156]. Among all the properties of boron nitride nanotubes (BNNTs), their excellent piezoelectricity is the most important for using them as nanovectors to deliver electrical or mechanical signals within cells [157].

#### 4.1.3. Zinc Oxide

Zinc oxide based piezoceramics are widely used due to their asymmetric hexagonal wurtzite structure and polar crystal surface. They have found application as piezoelectric nanogenerators, because of the easy fabrication [158]. ZnO in the shape of nanostructures is biocompatible [159]. It has been suggested that with ZnO size, its cytotoxicity increases, which has an influence on the levels of reactive oxygen species, reduces the mitochondrial membrane potential, and induces the production of interleukin in human cells. Additionally, it has been reported that chemical modification can reduce toxicity, providing a way for use in biomedical applications [160,161].

### 4.2. Piezopolymers

Piezoelectric polymers are a relatively new class of materials allowing the formation of electrical charges under mechanical stimulation in the absence of additional energy sources or electrodes [162]. Additionally, which is very important from the biomedical point of view, polymers are able to meet the requirements of biocompatibility and biodegradability, which is very crucial for new types of implants in regenerative medicine [163]. Further, their big advantage is the very high processing flexibility, which differentiates them from inorganic materials [164].

#### 4.2.1. Synthetic Polymers

##### Polyvinylidene Fluoride 

**Figure 8:**
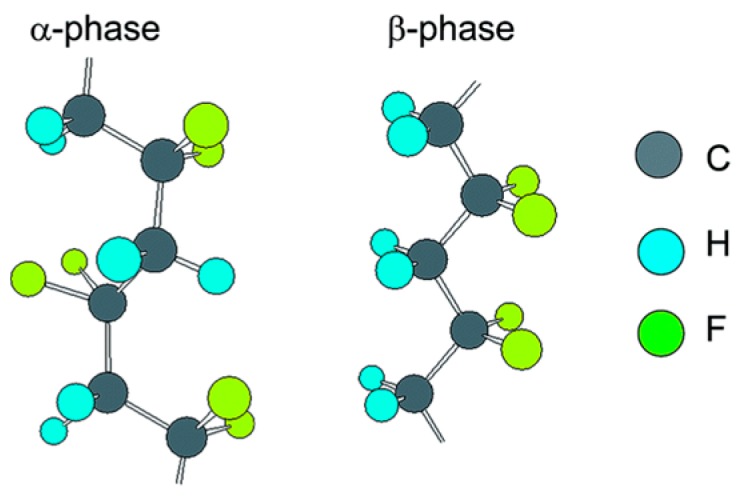
Structures of alpha and beta PVDF.

Among various piezoelectric polymers, PVDF is widely investigated, primarily because of its high piezoelectricity, processability, good chemical resistance, thermal stability, and good mechanical properties as compared to other piezoelectric polymers. PVDF may exist in at least five crystalline polymorphic phases, among which the β-phase shows the highest piezoelectricity, which reaches 20 pC/N (Figure 8) [165,166].

PVDF macromolecules may take various chain conformations and arrangement of CH_2_–CF_2_ molecular dipoles, resulting in various net dipole moments. A strong electric moment in the PVDF monomer unit arises from the strong electro-negativity of fluorine atoms as compared to hydrogen atoms. In the case that polymer chains are packed into crystals to form parallel dipoles, the crystal has a non-zero net dipole moment [89,90,91,92,93,94]. Such a molecular arrangement is observed in the β, γ, and δ phases, the first one showing the strongest dipole moment, due to the all-trans conformation. In the case of other chain conformations: TGTG- and T_3_GT_3_G-, parallel dipole moment arrangement, as in the δ and γ phases, respectively, leads to lower polarity; in the case of the same conformations, antiparallel chain dipole arrangement leads to the zero net dipole moment as in the α and ϵ phases [167].

The piezoelectricity of PVDF is phase content dependent, which hinges on the processing conditions. Obtaining a particular crystal phase is possible using various paths, involving melt or solution crystallization, annealing (also at high pressure), mechanical drawing, or electrical poling. The presence of polar phases is very important, in particular, due to its bioelectrical effect of the stimulation of the nervous system, holding promise for effective tissue regeneration [168,169].

As regards the most polar β-phase, it may be obtained, for example, by annealing at very high pressure from the α-phase, by poling at a very high electrical field from the α-phase or δ-phase [170] or drawing from the γ-phase [171]. In order to increase the content of polar phases, various methods are reported: melt-recrystallization [172], poling under a high electric field [173], application of high pressure [174], mechanical stretching [175], and the addition of nanoparticles, graphene, and nanowires [176]. 

The desire to use PVDF in piezoelectric scaffolds in tissue engineering requires the use of fabrication techniques that allow obtaining the proper morphology and high polar phase content responsible for the high piezoelectricity [177]. One of the promising fabrication techniques to fulfil both expectations is the electrospinning technique. Many publications were devoted to electrospinning of PVDF nanofibers from solution [178], determining the effect of processing parameters on the structure and properties of nanofibers and the characteristics of nonwoven nanofiber [179]. The content of the β-phase in PVDF was studied from the point of view of the applied voltage and rotation speed of the rotational collector. The collector rotational speed relates to the mechanical deformation, which is known to promote the formation of the polar phase [180]. Liu et al. [181] formed nanofibers with different rotational speeds of the collector: 900, 1100, 1300, 1500, 1700 and 1900 rpm. XRD diffraction showed a peak around 20.6–20.9 deg, which belonged to the β-phase, while the α-phase peaks disappeared. They received piezoelectric PVDF fibers with a small diameter, smooth surface morphology, and appropriate β-phase at a velocity of 1900 rpm. Recent works support the view that increasing of the rotational speed of the collector induces a higher content of the piezoelectric β-phase [182,183,184]. 

The hydrophobicity of PVDF is a problematic issue in neural tissue engineering. In order to reduce it, numerous research works have been conducted. In order to enhance the hydrophilic, as well as mechanical and electrical properties, PVDF has been modified by the addition of different nanostructures: nanoparticles [185,186,187], inorganic nanoparticles [188,189], nanotubes [190], and also by the addition of different polymers such as polyethylene glycol (PEG) [191] and polyvinyl alcohol (PVA) [192]. The addition of nanoparticles, especially metallic ones, improves the chemical, physical, and optical properties [193], while diamond nanoparticles have no significant impact on neuroblastoma cell morphology [194]. The incorporation of these nanostructures into a polymer piezoelectric scaffold can positively affect the nerve tissue. Additionally, modification of the surface can increase the neuron length and number of synaptic connections [195,196]. 

Arinzeh et al. [113,114] tested the piezoelectric scaffolds’ potential for promoting in vitro neural differentiation of human neural stem cells, thus demonstrating their applicability in neural tissue engineering. The authors in [197] extended the above studies by applying mechanical vibration, while generating electric fields to induce the piezoelectric effect in piezopolymers. The activation of the piezoelectric effect can be achieved by choosing various sources of mechanical stimulation, including vibration plates, sound, and ultrasound (US) [198,199]. Hoop et al. [85] investigated the influence of the piezoelectric PVDF substrate on supporting neural differentiation under dynamic stimulation. The results showed that the applied ultrasonic waves were sufficient to induce polarization in piezoelectric PVDF sheets and resulted in differentiation of PC12 cells. Piezoelectric PVDF can influence neuronal differentiation and neurite outgrowth of mouse neuroblastoma cells [200]. Electric fields have been shown to influence the growth and orientation of neurons in vitro, whereas the electric field was generated via electrodes [201]. Other studies have been reported successful neural stimulation in various piezoelectric systems, especially piezoelectric micro- and nano-fibers [202,203].

It was shown that the long term application of piezoelectric stimulation on neurons induces the number, length, and branching of neural cells with respect to non-stimulation conditions. No effects on neurite regeneration were observed when vibrations were applied to non-piezoelectric materials (e.g., mechanical stimulation of neurons) [204,205,206].

##### Poly-Vinylidene Fluoride-Trifluoroethylene 

Among the piezoelectric materials, this copolymer demonstrated the highest electroactive properties with a piezoelectric coefficient as high as 30 pC/N [207]. PVDF-TrFE forms the β-phase through copolymerization without the need for mechanical stretching or drawing [208]. In the case of additional annealing, mechanical stretching, or electrical poling, it is possible to further increase the crystallinity and alignment of the CF2 dipoles, thereby inducing higher piezoelectricity as compared to homopolymer PVDF, dependent on the TrFE content [209]. Electrospun PVDF-TrFE fibrous scaffolds showed higher crystallinity and β-phase content as compared to the starting powder material for neural and bone tissue engineering [101,102,103,104]. PVDF-TrFE and barium titanate piezoelectric composite membrane has been reported as a charge generator to promote bone regeneration [106].

PVDF-TrFE piezoelectric fibrous scaffolds were used to study their influence on neural repair. Many investigations reported a positive influence of PVDF-TrFE scaffolds on nerve cell growth and differentiation [210]. Lee et al. [114] fabricated a PVDF-TrFE piezoelectric electrospun scaffold with different orientations of the fibers, randomly and aligned. It was shown that the scaffold with aligned fibers had the highest potential in neural tissue engineering, especially in neurite outgrowth of dorsal root ganglion neurons. It was observed that PVDF-TrFE scaffolds can promote the formation of mature neural cells exhibiting neuron-like characteristics, while aligned fibers can promote primary neuron extension and can direct the neurite outgrowth [211]. 

Nerve guidance channels may be built using PVDF-TrFE for neural regeneration [212]. In this study, poled (negatively charged and positively charged) and unpoled channels were used. After four weeks, it was observed that the positively poled channels increased the number of regenerated nerves.

In muscle regeneration, the charge at the surface of PVDF films influences the cell proliferation [213]. However, until now, studies with specific dynamic conditions for piezoelectric PVDF-TrFE with mechanical or electrical stimulation have not been conducted.

##### Poly-3-Hydroxybutyrate-3-Hydroxyvalerate

PHBV is a polyester with a low piezoelectric coefficient (1.2 pC/N) [214]. It is a thermoplastic produced by many bacteria as an intracellular reservoir of carbon and energy. This polyester is also biodegradable, biocompatible, and exhibits strong mechanical properties, which allow using PHBV as a scaffold in biomedicine and as a biosensor [215,216]. PHBV has a comparable piezoelectric potential to that of bone, which can facilitate bone growth and healing [217,218]; thus, it can be used in the form of a composite with the addition of hydroxyapatite for bone tissue engineering. PHBV has been studied for neural tissue engineering, as a support for neuronal cell growth and axon dendrite polarization [219]. In the form of electrospun aligned PHBV fibers, with the addition of collagen, it can be used as a substrate for nerve tissue engineering [220,221,222].

##### Poly-L-Lactic Acid

Poly-L-lactic acid is a biodegradable and biocompatible polymer, with a piezoelectric coefficient of −10 pC/N [223]. Fukada et al. demonstrated that implantation of PLLA can promote bone growth in the response of its piezoelectric polarization [93]. PLLA with a structure similar to natural ECM may be used as a biomaterial in various biomedical applications [224]. Aligned PLLA nanofibrous scaffolds coated with graphene oxide promote neural cell growth [225]. Finally, the addition of iron oxide nanoparticles supports extending neurites along electrospun PLLA microfibers [226,227].

#### 4.2.2. Natural Biopolymers

Natural polymers are gaining more importance in tissue engineering because of their biodegradability and low toxicity. In general, many biopolymers exhibit piezoelectricity. As an example, we show some polysaccharides and proteins with relatively strong piezoelectricity.

##### Cellulose

Cellulose with a piezoelectric coefficient of 0.10 pC/N is a widely investigated natural polymeric material. Cellulose is a linear homopolymer of glucose with high biocompatibility [228,229,230,231,232,233,234]. It is used in different shapes and forms: membrane sponges, microspheres, and non-woven, woven, or knitted textiles. Cellulose has been investigated in the tissue engineering of bones [235,236], cartilage [237], for connective tissue formation [238], as a drug delivery system [239], and as a scaffold for growing functional cardiac cell constructs in vitro [240]. One of the important derivatives of cellulose is methylcellulose (MC), presenting in general good solubility in water, particularly at low temperatures, being dependent on the degree of methyl substitution and the distribution of methoxy groups. Collectively, these data indicate that MC is well suited as a biocompatible injectable scaffold for the repair of brain defects [241,242,243]. Gelatin coated nanoparticles contained in cellulose acetate/PLA scaffolds showed higher cell viability than uncoated scaffolds, and they acted as a nerve guidance conduit for sciatic nerve defects in vitro and in vivo [244], while a gelatin/chitosan/PEDOT hybrid scaffold enhanced the neurite growth of PC12 cells and promoted neuron-like cell adhesion and proliferation [245].

##### Chitin and Chitosan

Chitin is a natural polysaccharide with a piezoelectric structure with a low piezoelectric coefficient in the range from 0.2 to 1.5 pC/N [246]. It is a natural component of the cuticles of crustaceans, insects, and mollusks. Since it is hydrophilic and biocompatible, chitin is used for biomedical applications, promoting cell adhesion, proliferation, and differentiation [247]. 

Chitosan is a biodegradable and biocompatible linear polysaccharide obtained by partial deacetylation of chitin. It has been extensively investigated for the preparation of porous scaffolds for cartilage tissue engineering [248]. However, the low mechanical properties of scaffolds prepared from chitosan make its clinical application problematic. An effective method to overcome chitosan’s drawbacks is to blend it with synthetic polymers [249,250,251,252]. Skop et al. designed biocompatible chitosan microspheres for the delivery of neural stem cells and growth factors for CNS injuries [253]; another group designed chitosan particles loaded with the drug piperine, reported to have neuroprotective potential against Alzheimer’s disease, which successfully targeted specific areas of the brain [254]. Chitosan nanoparticles have also been developed for intranasal delivery of therapeutic agents to the brain [255,256]. Aligned PCL/chitosan fibers supported PC12 cells adhesion and growth, enhancing neurite extension along the fiber orientation [257]. PLGA/chitosan scaffolds guided neuronal differentiation for peripheral nerve regeneration both in vitro and in vivo [258,259].

##### Collagen

This is a natural piezoelectric material with a piezoelectric coefficient in the range from 0.2 to 2.0 pC/N [260]. Research has been reported on the application of collagen scaffolds in bone healing [261,262,263,264]. Furthermore, collagen-calcium phosphate composites have been reported for cartilage tissue engineering [265]. Similarly, collagen-hydroxyapatite piezoelectric composite scaffolds have been proven to be suitable for cellular growth [266]. Collagen scaffolds with the addition of chitosan have been tested in adipose tissue regeneration. Adipocytes were seeded, and the in vitro cytocompatibility and in vivo biocompatibility of scaffolds was confirmed experimentally [267]. An interesting application of collagen is entubulation, hence the use of magnetically aligned type I collagen gel, achieved by exposing the forming collagen gel to a high strength magnetic field, as a filler for collagen tubes. This method was successful in small peripheral nerve lesions, improving nerve regeneration significantly in a 6 mm nerve gap in mice [268] and guiding neurite elongation and Schwann cell invasion in vitro [269] and in vivo [270].

## 5. Conclusions and Future Perspectives

Recently, smart materials have been of great interest for scientists and physicians, because of the many opportunities to use them as candidates for developing the next generation of biomedical devices, transient implants, and drug delivery vehicles. Considering smart scaffolds for tissue repair and regeneration, piezoelectric materials have recently been of particular interest as they can deliver electrical stimulus without an external power source. There is no doubt that the bioelectric signals produced by piezoelectric scaffolds can regenerate and repair the tissues by definite pathways similar to the natural processes occurring within the natural extracellular matrix (ECM). The combination of morphology together with the chemical, mechanical, and electrical properties of the scaffolds is crucial for the success in tissue regeneration. Electrical charges are particularly important in neural tissue engineering, in which electric pulses can stimulate neurite directional outgrowth to fill gaps in nervous tissue injuries. There is no doubt that the perspective of the broader application of piezoelectric scaffolds as smart materials for neural tissue regeneration is of great importance, allowing avoiding traditional (invasive) electrical stimulation. It was shown recently in in vitro conditions that the deformation of the piezoelectric scaffolds either by mechanical or ultrasound stimulation led to neurite extension and enhanced cell adhesion and proliferation. However, one should be aware that most of the up-to-date experiments using piezoelectric scaffolds were performed without such stimulation, which does not lead to piezoelectricity and resulting electrical charges. In such a case, the only charges that can be active from the cellular perspective are surface charges due to permanent polarization, as well as related to transient deformation caused by the contraction and protrusion of the attached cells. Nonetheless, it is crucial from the perspective of experiments with piezoelectric scaffolds to mimic the in vivo conditions with internal macro- and micro-deformations by in vitro conditions using mechanical (ultrasounds) agitation, allowing obtaining a real piezoelectric response. The next problem in the area of piezoelectric scaffolds is related to the non-biodegradability of the polymers exhibiting the highest piezoelectric coefficients, i.e., PVDF and its copolymers. Therefore, attention should be focused on biodegradable piezoelectric polymers like PHB or PLLA. An interesting alternative, which should be explored in the future, is related to the composite scaffolds containing an electro-conductive polymer like PANi in addition to a piezoelectric polymer. It was shown that the addition of an electro-conductive polymer to the piezoelectric matrix resulted in an increase in piezoelectricity. This kind of composite scaffold should be taken into account, when thinking about biodegradable piezoelectric polymers with originally relatively low piezoelectricity.

## Figures and Tables

**Table 1:** Natural polymers with the piezoelectric response.

Natural Polymers		Piezoelectric Coefficient −d14 (pC/N)	Ref.
Collagen	Skin	0.2	[92]
	Bone	0.7	[92]
	Tendon	2.0	[93]
Keratin	Horn	1.8	[94]
	Wool	0.1	[94]
Fibrin	Salmon DNA	0.07	[93]

**Table 2:** Piezoelectric materials in nerve tissue engineering.

Material Type	Scaffold Design	Cells Type Used	Ref.
Polyvinylidene Fluoride (PVDF)	Film *	Spinal cord neurons	[104]
	Film *	Mouse neuroblastoma cells	[105]
	Channels	Mouse sciatic nerve model	[106]
	Tubes	Wistar rats	[107]
	Membranes	Neuronal cells	[108]
	Films	Stem cells	[109]
	Nanosheets *	Rat neuronal cell line	[110]
	Fibers *	Osteoblasts MG-63 cells	[111]
	Fibers	Mesenchymal stem cells	[112]
Poly[(vinylidene fluoride-co-trifluoroethylene] (PVDF-TrFE)	Fibers	Poietics normal human neural progenitors	[113]
		Dorsal root ganglion	[114]
	Films	Poietics normal human neural progenitors	[113]
	Membranes *	Osteoblasts SaOS-2 cells	[115]
	Tubes	In vivo implementation: rat sciatic nerves	[116]
	Fibers *	Preosteoblasts	[117]
Poly(3,4ethylenedioxythiophene) (PEDOT)	Films	Fibroblast growth factor (bFGF)	[118]
	Films *	-	[119]
	Films *	Neural stem cells	[120]
	Films	Neural stem cells	[121]
	Nanofibers *	Brain neuroglioma cells	[122]
Polylactic acid (PLLA)	Fibers	Sprague–Dawley rats	[123]
		PLLA blends for vascular differentiation in vitro	[124]
		Neural differentiation and growth in vitro	[125,126]
		PLLA blends for bone formation in vitro	[127]
	+PANi fibers *	Nerve stem cells	[128]
Poly(3-hydroxybutyrate-co-3-hydroxyvalerate) (PHBV)	Fibers *	Human mesenchymal stem cell	[129]
Collagen	Fibers	Schwann cells	[130]
	3D gel matrices	Embryonic rat cerebral cortices	[131]
BaTiO3	+PVDF matrix	Osteoblasts	[132]

* Tests conducted with electrical/mechanical stimulation.
